# Robotic assessment of rapid motor decision making in children with perinatal stroke

**DOI:** 10.1186/s12984-020-00714-1

**Published:** 2020-07-14

**Authors:** Rachel L. Hawe, Andrea M. Kuczynski, Adam Kirton, Sean P. Dukelow

**Affiliations:** 1 Department of Clinical Neurosciences, Hotchkiss Brain Institute, University of Calgary, 3330 Hospital Drive NW, Calgary, AB T2N 4N1 Canada; 2 Cumming School of Medicine, University of Calgary, 3330 Hospital Drive NW, Calgary, AB T2N 4N1 Canada; 3 Department of Pediatrics, Alberta Children’s Hospital, 2888 Shaganappi Trail NW, Calgary, AB T3B 6A8 Canada; 4 Alberta Children’s Hospital Research Institute, Alberta Children’s Hospital, 2888 Shaganappi Trail NW, Calgary, AB T3B 6A8 Canada

**Keywords:** Perinatal stroke, Hemiparetic cerebral palsy, Executive function, Robotics

## Abstract

**Background:**

Activities of daily living frequently require children to make rapid decisions and execute desired motor actions while inhibiting unwanted actions. Children with hemiparetic cerebral palsy due to perinatal stroke may have deficits in executive functioning in addition to motor impairments. The objective of this study was to use a robotic object hit and avoid task to assess the ability of children with hemiparetic cerebral palsy to make rapid motor decisions.

**Methods:**

Forty-five children with hemiparetic cerebral palsy due to perinatal stroke and 146 typically developing children (both groups ages 6–19 years) completed a robotic object hit and avoid task using the Kinarm Exoskeleton. Objects of different shapes fell from the top of the screen with increasing speed and frequency. Children were instructed to hit two specific target shapes with either hand, while avoiding six distractor shapes. The number of targets and distractors hit were compared between children with hemiparetic cerebral palsy and typically developing children, accounting for age effects. We also compared performance to a simpler object hit task where there were no distractors.

**Results:**

We found that children with hemiparetic cerebral palsy hit a greater proportion of total distractors compared to typically developing children, demonstrating impairments in inhibitory control. Performance for all children improved with age. Children with hemiparetic cerebral palsy hit a greater percentage of targets with each arm on the more complex object hit and avoid task compared to the simpler object hit task, which was not found in typically developing children.

**Conclusions:**

Children with hemiparetic cerebral palsy due to perinatal stroke demonstrated impairments in rapid motor decision making including inhibitory control, which can impede their ability to perform real-world tasks. Therapies that address both motor performance and executive functions are necessary to maximize function in children with hemiparetic cerebral palsy.

## Background

The defining feature of hemiparetic cerebral palsy (HCP) is motor deficits primarily on one side of the body. Our ability to interact with the world, however, depends on both motor skills and executive function. In order for a child to be able to ride a bike, they must not only have strength, coordination and balance, but they must also be able to make rapid decisions and execute actions quickly based on information integrated from the environment to navigate and avoid hitting obstacles. Early brain lesions can lead to impairments in both motor and executive function in children with HCP, significantly impacting a child’s ability to complete everyday activities [1].

Executive functions refer to the cognitive processes required for goal-directed behavior. Executive functions can be described as a group of three core functions: inhibition and interference control, working memory, and cognitive flexibility [2–4]. Executive functions underlie motor activities in everyday life. Environmental stimuli must be recognized and processed in order to make a decision about motor actions, which includes inhibiting unwanted movements [5, 6]. Executive functions are also critical for children with HCP to be able to compensate for motor impairments, incorporate the more affected arm in bilateral tasks, and achieve new motor skills through explicit learning [7, 8]. Beyond motor skills, executive function impairments can cause difficulty with academic performance [9], as well as general behavioral problems and hyperactivity [10].

Children with cerebral palsy have been reported to have impairments in attention and inhibitory control [11–16]. Most HCP is secondary to perinatal stroke, either arterial ischemic strokes (AIS) or periventricular venous infarcts (PVI) [17], with disease-specific studies showing frequent impairments in executive function [18–21]. Compared to other periods of development, lesions acquired during the perinatal period may present a higher risk for impairments in executive functions [21]. This may relate to the developing brain having more widespread representations of executive functions than the mature brain [22]. While frontal and pre-frontal regions are responsible for executive functions in adults, damage to more diverse areas of the developing brain can impede ongoing and future development of executive functioning [11, 22]. Additionally, since executive functions mature through experience in typical development, children with HCP who may have decreased participation in activities with peers [23] may have slowed or decreased concurrent cognitive development [11].

Much of the previous work on executive function impairments in children with cerebral palsy has focused on neuropsychological assessments and has not examined executive function in terms of a motor task [12, 14, 15]. Additionally, assessments of motor impairments in children with cerebral palsy use a standardized environment and minimize potential distractions, thus not examining how executive function may impact motor performance [24, 25]. As both executive and motor function impairments have been established in children with hemiparetic cerebral palsy, it is imperative to understand how they may interact in the context of a more complex motor task. Additionally, as lesion type (AIS vs. PVI) can impact the brain differently, we must understand how impairments differ between stroke types. Overall, understanding the interplay of executive function impairments and motor impairments will aid clinicians in understanding and addressing challenges children may face in real world tasks.

In this study, we used a bilateral robotic object hit and avoid task that required participants to make rapid motor decisions and actions to hit certain shapes and avoid others [26]. Robotic assessments of motor, sensory, and visuospatial attention have been previously used in typically developing children and children with hemiparetic cerebral palsy and are well-tolerated, feasible, and safe [27–30]. Using a robotic task, we can combine motor and executive function assessments in a controlled manner with a level of quantification not possible with traditional clinical assessments. The present study builds on a previous study that used a robotic object hitting task [30] but did not involve distractor objects. The object hit and avoid task requires participants to remember the specific shapes of the target objects, attend to multiple moving objects across the workspace, quickly determine if they are a target or distractor, and execute the motor plan (hit or avoid) accordingly. Our aims were to: 1) determine impairments in the ability of children with HCP due to perinatal stroke (AIS or PVI) to make rapid motor decisions and actions in an object hit and avoid task; 2) examine the effect of age on task performance; 3) determine if the increased cognitive complexity of the task negatively impacts goal-directed behavior (ability to hit the targets) compared to a simpler version of the task that does not have any distractors. We hypothesized that children with HCP would hit a greater proportion of distractors compared to typically developing children. Additionally, we hypothesized that the added complexity of the task would cause children with HCP to hit a lower percentage of targets compared to the simpler object hit task.

## Methods

### Participants

A convenience sample of participants with perinatal stroke and hemiparetic cerebral palsy confirmed by MRI [17] were recruited from a population-based cohort (Alberta Perinatal Stroke Project) [31]. Inclusion criteria were: 1) age 6 to 19 years old; 2) unilateral perinatal stroke (arterial ischemic stroke (AIS) or periventricular venous infarct (PVI)) confirmed by MRI; 3) clinical confirmation of symptomatic hemiparesis; 4) born at gestational age > 36 weeks; 5) visual acuity of at least 20/50 corrected; and 6) written informed consent/assent. Exclusion criteria were: 1) multifocal stroke; 2) other neurological disorders not attributable to perinatal stroke; 3) Manual Abilities Classification System grade V; 4) severe spasticity demonstrated by Modified Ashworth Scale > 3 in the upper extremity; 5) inability to comply with the study protocol; 6) upper extremity surgery, botulinum toxin treatment, constraint induced movement therapy, or brain stimulation therapy within 6 months of study participation. A convenience sample of typically developing (TD) children between the ages of 6 and 19 with no history of neurologic impairment were also recruited from the general population. Participants in the TD cohort were recruited to ensure our sample was distributed across the age range and balanced between males and females. Participants and/or parents provided written informed assent/consent. This cohort of TD children and children with HCP completed a battery of sensory, motor, and visuospatial robotic assessments that have been previously published [27–30]. The study was approved by the University of Calgary Conjoint Health Research Ethics Board.

### Robotic task

**Fig. 1:**
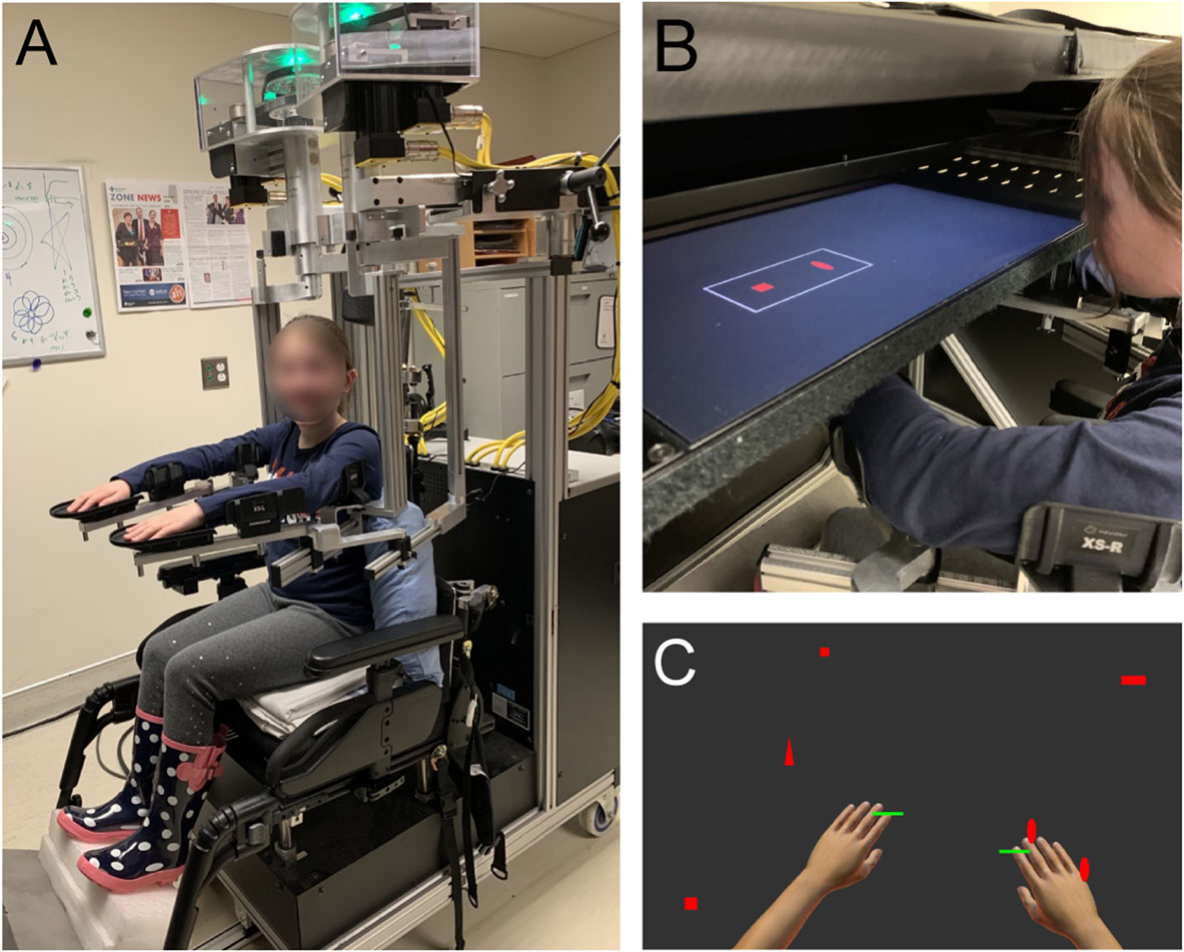
Robotic Object Hit and Avoid Task. **a** Participant is shown seated in a modified wheelchair base with their arms supported in the horizontal plane by troughs adjusted to allow free movement at the shoulders and elbows. **b** Participants are wheeled into the augmented reality setup. Displayed on the horizontal screen are the two target shapes that the participant is instructed to hit. **c** The workspace of the object hit and avoid task is shown. Note that during the task, participants do not see their hands but only see the green paddles located at their fingertips

Participants performed an object hit and avoid task using the Kinarm exoskeleton robot (Kinarm, Kingston, ON, Canada) where applications in this population are well established [27–30]. Participants sat in a wheelchair base modified to fit pediatric participants, with their arms supported in the horizontal plane, as shown in Fig. 1a and b. The Kinarm linkages were adjusted to allow for free motion at the shoulders and elbows in the horizontal plane. Participants’ vision of their limbs was occluded using a shutter. Virtual reality was provided through a horizontally mounted display. Children were able to familiarize themselves with moving their arms in the virtual environment prior to starting data collection, though specific practice trials on the task were not provided.

At the start of the task, participants were shown two shapes that would serve as the targets, such as a circle and square, and instructed to only hit those shapes in the same orientation while avoiding all other shapes or orientations (distractors). Participants were allowed to view the target shapes for as long as needed before proceeding with the task. During the task, 5 cm paddles were displayed at the participant’s fingertips bilaterally, and they were instructed to use the paddles to hit the target shapes away from them towards the top of the screen. Participants could select which hand to use to hit each target. Objects were released from the top of the screen from 10 different horizontal locations. During the task, the speed and frequency of the objects increased. A total of 200 targets and 100 distractors were dropped. The task lasted 138 s. The task has been previously described in detail for application in adult stroke [26]. The workspace for the task can be seen in Fig. 1c.

Performance on the task was measured and analyzed using the following parameters. Note that we are using the terms “dominant” and “non-dominant” to apply to both children with HCP and TD children. In children with HCP, dominant refers to the less affected (ipsilesional) arm and non-dominant refers to the more affected (contralesional) arm.

1. *Total Target Hits:* Total number of targets hit.
2. *Target Hits Non-dominant:* Number of targets hit with non-dominant hand.
3. *Target Hits Dominant:* Number of targets hit with dominant hand.
4. *Total Distractor Hits:* Total number of distractors hit.
5. *Distractor Hits Non-dominant:* Number of distractors hit with non-dominant hand.
6. *Distractor Hits Dominant:* Number of distractors hit with dominant hand.
7. *Distractor Proportion:* The number of distractors hit as proportion of total objects (targets and distractors) hit. This accounts for fewer overall objects being hit in HCP due to motor deficits.
8. *Object Processing Rate:* The rate of objects correctly being processed (number of targets hit and distractors being missed) per second at 80% task completion [26].

Performance on the object hit and avoid task was also compared to a simpler object hit task that was always completed immediately before the object hit and avoid task in the same session. The object hit task has 300 targets (all circles) and no distractors, and participants were instructed to hit as many targets as possible in the 138 s. The object hit task in children with HCP has been previously described in detail [30].

### Clinical measures

Clinical measures were also collected on participants with HCP by a trained therapist. The conventional subset of the Behavioral Inattention Test, a pencil and paper test of spatial attention including line bisection, line/letter/star cancellation, figure copying and drawing, was used with a maximum score of 146 and scores below 130 indicative of visuospatial neglect [32]. The Melbourne Assessment was used as an assessment of reaching and grasping function in children with HCP with scores ranging from 0 (worst) to 100 (best) [24]. The Assisting Hand Assessment [33] was used to assess use of the affected arm and hand in bimanual tasks with scores converted to a logit scale ranging from 0 (no use) to 100 (full use) of the affected arm.

### Statistical analysis

One-way ANCOVAs accounting for age were used to determine group (TD vs. AIS vs. PVI) mean differences for each parameter. Levene’s test was used to test for homogeneity of variances. Standardized residuals for each group and the overall model were assessed for normality using the Shapiro-Wilk’s test, as well as visual inspection. The assumption that residuals are normally distributed was violated for the TD group and overall model fit for total distractors, hits non-dominant, distractor hits non-dominant, and distractor hits dominant. Considering that ANCOVAs are robust to violations of normality, and visual inspection demonstrated the residuals approximated a normal distribution, we proceeded with analysis. When significant group differences were found, post-hoc t-tests were used to make pairwise comparisons using Bonferroni corrections (*p* < 0.0167). To determine impairments on an individual rather than group level, age-based curves (second order polynomials) were fit to the data from the TD participants and 95% prediction bands were calculated. Children were identified as impaired if they fell outside the 95% bands. The percentage of children in the AIS and PVI groups that were identified as impaired were calculated. For the remaining analyses, AIS and PVI were combined as we were more interested in the differences between TD and HCP rather than differences between stroke types. Regression analyses were conducted to determine the impact of age on each parameter for TD and HCP. To compare performance between the object hit task and the object hit and avoid task, the number of hits with each hand as a percentage of total targets (300 for object hit and 200 for object hit and avoid) were calculated. Paired t-tests were then used to compare the percentage of hits between tasks, and Cohen’s d effect sizes were calculated. Effects of 0.2 were considered small, 0.5 to be medium, and 0.8 to be large [34]. Parameters from the robotic object hit and avoid task were correlated with clinical measures using Pearson correlations. For all analysis, *p*-value < 0.05 was considered significant. Statistical analyses were performed using SPSS version 24 or Matlab 2018a.

## Results

Overall, 45 children with HCP and 146 TD children completed the study. Participant demographics are shown in Table 1. No differences in age were found between groups (F (2,188) = 1.001, *p* = 0.37). Of the clinical scores (BIT, AHA, and Melbourne), the AIS and PVI groups were only found to be significantly different for the Melbourne, in which the PVI group scored higher than the AIS group (t (27) = − 2.50, *p* = 0.0187).

**Table 1:** Demographic and Clinical Characteristics

	TD	AIS	PVI
*N*	146	26	19
*Male/Female*	77/69	17/9	13/6
*Affected Arm*	Handedness: 12 L/134 R	8 L/ 18 R	10 L/9 R
*Age (mean years ± SD)*	13.0 ± 4.1 range: 6.1–19.9	12.8 ± 4.0 range: 6.4–19.5	11.6 ± 3.7 range: 6.6–18.5
*MACS*		MACS I: *N =* 4, MACS II: *N* = 14	MACS I: *N* = 7 MACS II: *N* = 4
*BIT (mean ± SD)*		132 ± 17.8	138.6 ± 5.4
		range: 59–145	range: 122–146
		*N* = 5 below cutoff for neglect (< 130)	*N =* 1 below cutoff for neglect (< 130)
*AHA (mean ± SD)*		62.4 ± 21.3	73.4 ± 16.2
		range: 32–100	range: 55–100
*Melbourne (mean ± SD)*		69.6 ± 23.0	88.4 ± 11.7
		range: 31–100	range: 64–100

*MACS* Manual Ability Classification Scale*, BIT* Behavioral Inattention Test, *AHA* Assisting Hand Assessment*.*

*MACS, AHA, and Melbourne scores were unavailable for 16 children (8 AIS, 8 PVI), and BIT scores were unavailable for 1 child with AIS.*

### Group differences

Group means adjusted for age and results of the ANCOVAs and post-hoc pair-wise comparisons are shown in Table 2. For most parameters, both the AIS and PVI groups performed worse (fewer targets, more distractors) compared to the TD group, however, the AIS and PVI groups were only different from each other for total targets hit and target hits with non-dominant hand.

**Table 2:** Group Means and Between-Group Comparisons

		Mean (St. Err)	Group Differences (df = 2, 187)	TD vs. AIS (df = 170)	TD vs. PVI (df = 163)	AIS vs. PVI (df = 43)
Total Target Hits	TD	127 (1.29)	***F =*** **50.7,*p <*** **0.0001**	**t = 9.66,*p <*** **0.0001**	**t = 4.09,*p <*** **0.0001**	**t = −3.49,*p*** **= 0.001**
	AIS	94.8 (3.06)				
	PVI	111.3 (3.60)				
Target Hits Non-Dominant	TD	61.0 (0.84)	***F =*** **84.1,*p <*** **0.0001**	**t = 11.93,*p*** **< 0.0001**	**t = 6.63,*p*** **< 0.00001**	**t = − 3.02,*p*** **= 0.004**
	AIS	35.3 (1.98)				
	PVI	44.6 (2.33)				
Target Hits Dominant	TD	65.9 (0.86)	***F =*** **4.6,*p*** **= 0.012**	**t = 2.94, *p*= 0.0038**	t = −0.31, *p* = 0.76	t = −2.32, *p* = 0.025
	AIS	59.5 (2.0)				
	PVI	66.7 (2.4)				
Total Distractor Hits	TD	15.6 (0.64)	**F = 10.6,*p <*** **0.0001**	**t = −3.44,*p*** **= 0.0007**	**t = −3.50,*p*** **= 0.0006**	t = −0.41, *p* = 0.68
	AIS	21.3 (1.53)				
	PVI	22.2 (1.79)				
Distractor Hits Non-dominant	TD	7.8 (0.34)	*F =* 1.4, *p* = 0.251	–	–	–
	AIS	8.9 (0.79)				
	PVI	9.1 (0.93)				
Distractor Hits Dominant	TD	7.8 (0.39)	***F =*** **18.7,*p*** **< 0.0001**	**t = −4.56,*p*** **< 0.0001**	**t = −4.63,*p*** **< 0.0001**	t = −0.54, *p* = 0.59
	AIS	12.4 (0.93)				
	PVI	13.2 (1.10)				
Distractor Proportion	TD	11.3 (0.43)	***F =*** **33.0,*p <*** **0.0001**	**t = −6.86,*p*** **< 0.0001**	**t = −5.22,*p <*** **0.0001**	t = 0.59, *p* = 0.56
	AIS	18.8 (1.01)				
	PVI	17.9 (1.18)				
Object Processing Rate	TD	1.97 (0.019)	***F =*** **40.3,*p <*** **0.0001**	**t = 8.28,*p <*** **0.0001**	**t = 4.51,*p <*** **0.0001**	t = −2.23, *p* = 0.031
	AIS	1.55 (0.046)				
	PVI	1.71 (0.05)				

*Group means adjusted for age and results of ANCOVAs and post-hoc pairwise comparisons are shown. Results are bolded if significant (p < 0.05 for ANCOVAs or p < 0.0167 for post-hoc tests (Bonferroni Correction)).*

*TD* typically developing, *AIS* arterial ischemic stroke, *PVI* periventricular venous infarct.

### Percentage of groups with impairments

**Fig. 2:**
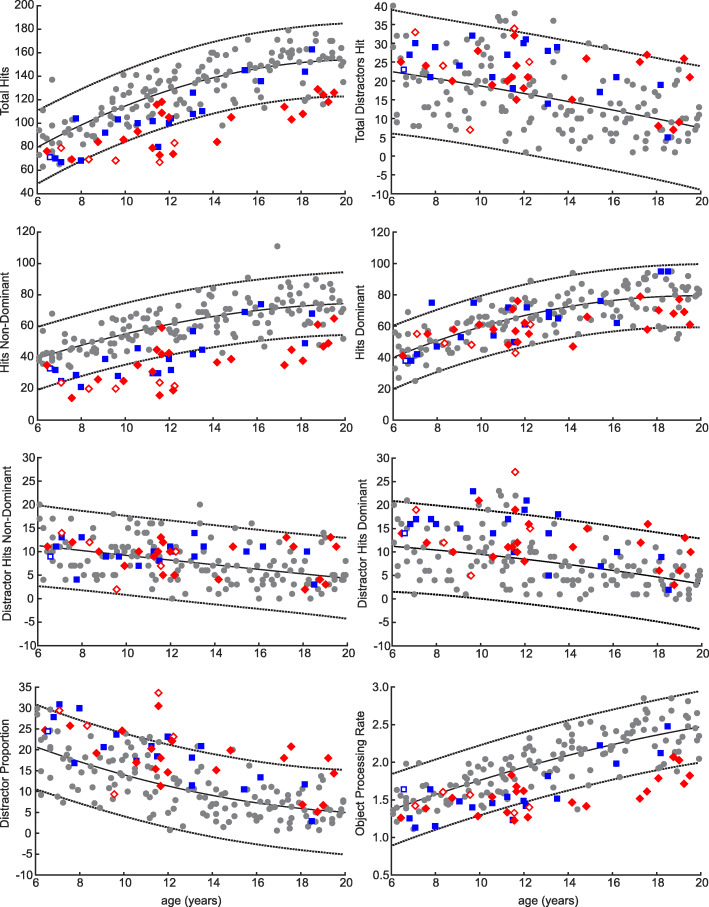
Age Curves: Age curves are shown for each parameter. Curves were fit to typically developing (TD) participants, with individual participants displayed with grey circles and curves with 95% prediction bands shown in the black solid and dashed lines respectively. AIS and PVI participants are superimposed in red and blue. Participants identified as having visuospatial neglect (< 130 on the BIT) are shown as open shapes. Participants falling outside the prediction bands are considered impaired

**Fig. 3:**
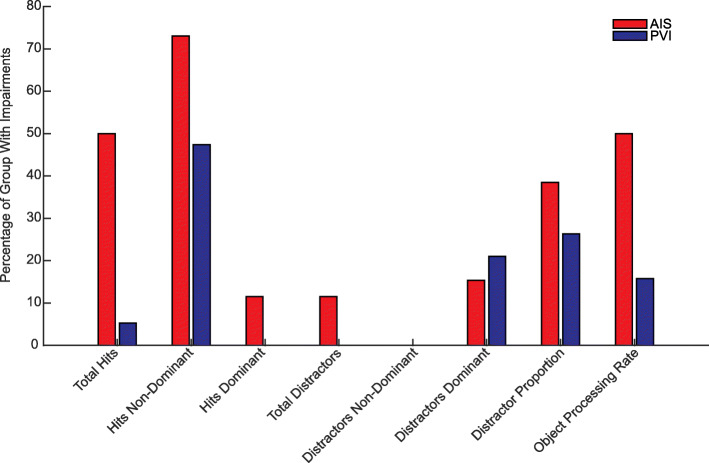
Proportions of impairment by stroke type. Percentage of the AIS and PVI groups that were identified as impaired based on the 95% prediction bands for TD children

Children were identified as impaired if their performance fell outside the 95% prediction bands for their age based on TD children, as shown in Fig. 2. Children who were found to have visuospatial neglect based on BIT scores below 130 are depicted in Fig. 2 as open shapes. The percentage of the AIS and PVI groups identified as impaired on each parameter are shown in Fig. 3. Notably, 73% of AIS and 47% of PVI were impaired on hits with the non-dominant hand. Rates of impairments on total distractors and distractor hits with each hand were low, however, distractor proportion, which accounts for the fewer number of total objects (targets and distractors) hit by children with HCP identified 38.5% of AIS and 26.3% of PVI as impaired. Additionally, the object processing rate identified 50% of AIS and 15.8% of PVI as impaired.

### Effect of age on performance

Regression analyses were completed to determine the role of age on task performance in TD participants and participants with AIS and PVI. Results are shown in Table 3. With the exception of distractors hit with the non-dominant arm, the number of targets hit increased and distractors decreased with age for both TD and HCP groups.

**Table 3:** Regression Analysis of Age and Task Performance

		TD	HCP
Total Hits	β	5.27	4.710
	p	< 0.001	< 0.001
	R^2^	0.644	0.575
Hits Non-Dominant	β	2.48	2.550
	p	< 0.001	< 0.001
	R^2^	0.507	0.443
Hits Dominant	β	2.79	2.160
	p	< 0.001	< 0.001
	R^2^	0.549	0.392
Distractors Total	β	−1.070	−0.810
	p	< 0.001	0.003
	R^2^	0.230	0.187
Distractors Non-Dominant	β	−0.496	−0.194
	p	< 0.001	0.133
	R^2^	0.190	0.052
Distractors Dominant	β	−0.576	−0.618
	p	< 0.001	0.002
	R^2^	0.200	0.202
Distractors Proportion	β	−1.110	−1.230
	p	< 0.001	< 0.001
	R^2^	0.452	0.441
Object Processing Rate	β	0.079	0.051
	p	< 0.001	< 0.001
	R^2^	0.656	0.443

*Results of regression analyses examining relationship between age and performance on each parameter are shown with coefficient for age (β), significance (p) and variance explained (R*^*2*^*).*

*TD* typically developing*, HCP* hemiparetic cerebral palsy*.*

### Effect of task complexity

Performance in terms of percentage of targets hit was compared between the object hit and avoid task and a simpler object hit task without distractors to determine the effect of increasing task complexity. For the non-dominant arm in TD participants, there was no difference in the percentage of targets hit between object hit and object hit and avoid (30.5 ± 7.5% vs. 30.7 ± 7.2%), *p* = 0.44, Cohen’s d = 0.07). For the dominant arm in TD, participants hit a higher percentage of the targets in object hit compared with object hit and avoid (34.6 ± 8.0% vs. 33.2 ± 7.7%, *p* < 0.001, Cohen’s d = 0.34 (small effect size)). For the non-dominant arm in HCP, participants hit fewer targets in object hit compared with object hit and avoid (17.7 ± 6.4 vs. 18.9 ± 7.5%, *p* = 0.022, Cohen’s d = 0.33 (small effect size)). For the dominant arm in HCP, participants also hit fewer targets in object hit than in object hit and avoid (27.4 ± 6.2% vs. 30.5 ± 6.7%, *p* < 0.001, Cohen’s d = 0.86 (large effect size)).

### Correlations with clinical scores

Correlations between task performance and clinical assessments are shown in Table 4. The Behavioral Inattention Test was correlated with all task parameters except distractor hits non-dominant and object processing rate. The Melbourne Assessment was correlated with measures of targets hit, but not parameters associated with distractors. The AHA significantly correlated with only total hits and hits non-dominant but none of the distractor parameters.

**Table 4:** Correlations Between Clinical Measures and Task Performance

	Behavioral Inattention Test	Melbourne Assessment	Assisting Hand Assessment
**Total Hits**	***r =*** **0.49,*p*** **= 0.0006**	***r =*** **0.45,*p*** **= 0.015**	***r =*** **0.39,*p*** **= 0.038**
**Hits Nondominant**	***r =*** **0.43,*p*** **= 0.004**	***r =*** **0.40,*p =*** **0.03**	***r =*** **0.43,*p*** **= 0.020**
**Hits Dominant**	***r =*** **0.42,*p*** **= 0.005**	***r =*** **0.38,*p*** **= 0.04**	*r =* 0.24, *p* = 0.211
**Total Distractors**	***r =*** **− 0.31,*p =*** **0.04**	*r =* 0.006, *p* = 0.97	*r =* −0.12, *p* = 0.52
**Distractors Nondominant**	*r =* −0.03, *p* = 0.85	*r =* −0.05, *p* = 0.81	*r =* − 0.09, *p* = 0.66
**Distractors Dominant**	***r =*** **−0.41,*p*** **= 0.006**	*r =* 0.04, *p* = 0.84	*r =* −0.12, *p* = 0.54
**Distractor Proportion**	***r =*** **−0.50,*p =*** **0.005**	*r =* −0.20, *p* = 0.30	*r =* − 0.27, *p* = 0.16
**Object Processing Rate**	*r =* 0.28, *p* = 0.062	*r =* 0.24, *p* = 0.22	*r =* 0.27, *p =* 0.16

*Bolded values indicate the correlation was significant (p < 0.05).*

## Discussion

The aim of this study was to examine the performance of children with HCP secondary to perinatal stroke on a bilateral rapid motor decision and action task. This robotic task combined motor skills with executive function. Children must keep the target shapes in their working memory while completing the task. They must be able to attend to different moving objects, and perform the necessary decision making to determine if the object is a target or distractor. Lastly, they must act accordingly, either hitting the moving target or exhibiting inhibitory control to not hit the distractors. Overall, we found that children with HCP hit fewer targets and more distractors than typically developing children, demonstrating impairments in both motor performance and executive function. We did not find group differences between stroke types (AIS vs. PVI) in any parameter related to hitting distractors. We found age to be related to task performance for both typically developing children and children with HCP. We also found that the added complexity of the task did not negatively impact the ability of children with HCP to hit the targets.

Hits with the non-dominant hand identified the greatest number of children as impaired based on falling outside the 95% prediction bands. This is not surprising since hitting the targets requires both motor skill and executive function. Compared to the 73% of children with AIS and 47% of children with PVI who had impairments on targets hit with the non-dominant arm, far fewer were identified as being impaired on distractor proportion (38% of AIS and 26% of PVI). However, despite only a minority of children being outside the range of TD children, there were significant group-level differences between children with HCP and TD. Examination of Fig. 2 also shows that while many children with HCP fall within the normal range for distractor proportion, they trend towards the higher end (more distractors) of normal. Borderline or mild impairments in executive function may impede a child’s function when coupled with motor impairments.

An interesting finding was that differences between AIS and PVI groups existed for total targets hit and targets hit with the non-dominant hand, but not for parameters relating to distractors. For targets hit, the AIS group had more significant impairments than the PVI group, which is consistent with previous studies [27–29, 35]. The lack of differences between AIS and PVI groups for parameters relating to distractors may suggest that executive function is not different between lesion types. Since executive functions have widespread representations in the developing brain, both types of lesions may impact their continued development [12, 22].

Our findings show that task performance improves with age in both TD children and children with HCP. Age-related changes in TD children are expected based on the maturation of both motor skills and executive functions, which have been shown to develop in concert with each other [6, 36–44]. There has not previously been a good understanding of the role of age on executive functions in children with HCP due to small samples of limited ages. Our findings are promising that executive functions do improve with age, though unfortunately the gap between TD and HCP may widen rather than decrease. The increasing gap is apparent when examining the age curves for Object Processing Rate (Fig. 2), where children with HCP do not start to fall outside of the normative ranges (prediction bands) until after age 11. Future research on the development of executive functions utilizing longitudinal designs is warranted.

A surprising finding that contradicted our hypothesis was that the added complexity of the object hit and avoid task compared to a simpler object hit task did not negatively impact the ability of children with HCP to hit the targets. Children with HCP actually hit a greater percentage of the targets in the object hit and avoid task than the simpler object hit task. There are several possible explanations for this. First, while the object hit and avoid task had greater complexity, the overall number of targets was lower (200 vs. 300), thus there are fewer targets to hit which might benefit children with motor impairments. However, given that no TD developing children were at the ceiling of targets hit in the object hit task, they would also be expected to benefit from having fewer targets to hit in the object hit and avoid task. Second, the object hit and avoid task was always done after the object hit task. Children with HCP may benefit from the practice of completing the simpler object hit task more than TD children. Lastly, there is a possibility that the added cognitive load improved or focused participant’s attention, resulting in better performance. Further work is needed to determine the reason behind the improved performance with added task complexity.

While it is important to study executive function in the context of a complex motor task, this can make it difficult to decipher the underlying cause of impaired behavior. A distractor may be hit due to the participant being unable to quickly determine if it is a target or distractor; determine the object is a distractor but lack the inhibition to not hit it; or lack the motor capability to move out of the way of the distractor due to motor impairments including weakness [45] and spasticity [46, 47]. It is unlikely that children with HCP hit a greater proportion of distractors due to motor impairment, as the non-dominant arm was not found to have an increase in number of distractors hit compared to TD children. If the deficit was in the processing and classifying of objects as targets or distractors, we would expect the number of targets to be affected as well as distractors. However, we found children with HCP actually hit more targets when having to decipher target or distractor than in the simpler object hit task where there were no distractors. The increase in distractors hit is therefore likely due to deficits in inhibitory control in children with HCP, which is consistent with prior literature [12, 14].

This study has several limitations. We did not have formal neuropsychological testing on our participants, which would help to better understand the relationship between specific executive functioning impairments and performance on the task. It is also possible that children in the TD group had executive function impairments that were not diagnosed. The simpler object hit task was always done before the object hit and avoid task, which may create a learning effect that could impact the TD and HCP groups differently. A longitudinal design would be preferential to our cross-sectional design to explore age effects and developmental changes in executive functions. Care should be taken in generalizing the findings of impairments on this task to impairments in inhibitory control in a broader sense, such as general behavioral issues. Additionally, in terms of generalizing findings, our participants may not represent the range of executive function impairments in children with HCP in general, as participants had to be able to follow instructions and understand the robotic tasks. Therefore, we are likely missing a subset of children with more severe impairments.

Our findings demonstrate that children with HCP have impairments in executive function, specifically inhibitory control, which impacts performance on a complex motor task. However, we find that despite deficits in inhibitory control, overall performance (i.e. hitting targets) did not degrade with the added complexity of the task but actually improved. Our findings have several implications for rehabilitation. Assessments and interventions for children with HCP typically focus on reach and grasp, with therapists often setting up the environment to optimize success by removing distractions. Environments with varying levels of complexity should be used in assessing motor impairments to understand how complex environments may impact (for better or worse) motor performance. Since impairments in executive function may compound motor impairments, rehabilitation should progress the environmental complexity to promote development of executive functions in parallel with motor skills. This will help the child execute motor actions in situations requiring rapid processing and inhibitory control, including sports, video games, and driving. As inhibitory control deficits impact both arms and bimanual tasks, complex motor tasks with the dominant arm requiring executive functions can also be a target of rehabilitation. Lastly, since executive functions develop in part due to experiences, children should be encouraged to participate in a range of age-appropriate activities with their peers, and therapists should address any barriers that may limit these key developmental experiences.

## Conclusions

In conclusion, we found that children with HCP due to perinatal stroke have an impaired ability to make rapid motor decisions and actions. Deficits in inhibitory control may impede a child’s ability to generate the appropriate movements in response to their environment. Therapies should address impairments in both motor skills and executive functions to maximize children’s functional outcomes.

## Data Availability

The data used and analyzed in the current study are available from the corresponding author on reasonable request.

## Abbreviations

AHA: Assisting Hand Assessment
AIS: Arterial Ischemic Stroke
BIT: Behavioral Inattention Test
HCP: Hemiparetic Cerebral Palsy
MACS: Manual Ability Classification Scale
PVI: Periventricular Venous Infarction
TD: Typically Developing
